# Editorial: Advances in brain functional and structural networks modeling *via* graph theory

**DOI:** 10.3389/fnins.2022.1031280

**Published:** 2022-11-03

**Authors:** Farras Abdelnour, Amy Kuceyeski, Ashish Raj, Yasser Iturria-Medina, Samuel Deslauriers-Gauthier

**Affiliations:** ^1^University of California, San Francisco, San Francisco, CA, United States; ^2^Department of Radiology, Weill Cornell School of Medicine, New York, NY, United States; ^3^Department of Radiology, University of California, San Francisco, San Francisco, CA, United States; ^4^Neurology and Neurosurgery, Ludmer Center, McConnell Brain Imaging Centre, McGill University, Montreal, QC, Canada; ^5^Research Centre Inria Sophia Antipolis Méditerranée, Université Côte d'Azur, Nice, France

**Keywords:** graph theory, brain networks, brain modeling, brain disease, structural network, functional network

The past decade has witnessed a great interest within the neuroscience community in modeling the function and structure of the brain and probing the dynamics between the two. DTI has been widely used to extract the structural network of the brain while the functional network has often been obtained *via* fMRI. Both linear and non-linear models predicting the functional network from the structural one have been proposed. Such models potentially offer valuable tools in identifying abnormal regions of the brain.

The aims of this Research Topic are to advance the current understanding of the brain modeled as a mathematical network, develop new and advanced methods capturing the graph relationship between function and structure, propose new analysis methods of brain time series explicitly grounded on underlying structural network, and ultimately apply the resulting knowledge to brain disease and further foster the understanding of the brain's underlying configuration and dynamics. The following articles touch on the latest headways in brain network research.

*Network analysis of time series: Novel approaches to network neuroscience by* *Varley and Sporns*.

There has been an explosion of interest in modeling the brain as a network, now referred to as “network neuroscience.” The authors propose network-based analysis of non-linear time series and review applications of these methods to neural data. Instead of preserving spatial information and collapsing across time, network analysis of time series collapses spatial information, preserving temporally extended dynamics. This allows researchers to infer “intrinsic manifold” from empirical brain data. The authors show how techniques from network science, non-linear dynamics, and information theory can extract meaningful information distinct from what is normally accessible in standard network neuroscience approaches.

*Brain functional connectivity analysis in patients with relapsing-remitting multiple sclerosis: A graph theory approach of EEG resting state by* *Shirani and Mohebbi*.

This study investigates the MS effects on the brain's functional connectivity network using EEG signals and graph theory. The objective is to assess the differences in brain functional network global features. The results demonstrate lower cortical activity in the Alpha and higher activity for the Gamma frequency bands in RRMS patients. Graph metric calculations reveal a significant difference in the diameter of the functional brain network, indicating a higher diameter in RRMS cases for the Alpha frequency band. Considerable differences between the networks' global efficiency and transitivity based on coherence measure are observed. The study indicates that in RRMS cases, some of the global characteristics of the brain's functional network change and can be illustrated even in the resting state condition when the brain is not under cognitive load.

*Predicting functional connectivity from observed and latent structural connectivity via eigenvalue mapping by* *Cummings et al*.

Understanding how complex dynamic activity propagates over a structural network is an overarching question in neuroscience. Previous work has demonstrated that linear graph models perform as well as non-linear neural simulations in predicting functional connectivity with the added benefits of low dimensionality and a closed-form solution. The authors propose a relating the eigenvalues of the structural connectivity and functional networks using the Gamma function, producing a prediction of functional connectivity with a single model parameter. They additionally investigate the impact of local activity diffusion and long-range interhemispheric connectivity on the structure-function model and show an improvement in functional connectivity prediction when accounting for latent variables.

*Brain network topology and structural–functional connectivity coupling mediate the association between gut microbiota and cognition by* *Zhang et al*.

Increasing evidence indicates that gut microbiota can influence cognition *via* the gut–brain axis, and brain networks play a critical role during the process. Little is known about how brain network topology and structural–functional (SC-FC) connectivity coupling contribute to gut microbiota-related cognition. 3-Back, digit span, and Go/No-Go tasks were employed to assess cognition. The authors test for potential associations between gut microbiota, complex brain networks, and cognition. They show that gut microbiota can affect the global and regional topological properties of SC and FC. They note that causal mediation analysis validate that gut microbial diversity and enterotypes indirectly influence cognitive performance by mediating the small-worldness of SC and some nodal metrics of FC. Their findings reveal novel insights which are essential to provide the foundation for previously unexplored network mechanisms in understanding cognitive impairment.

*Disrupted brain functional network topology in essential tremor patients with poor sleep quality by* *Peng et al*.

Poor quality of sleep (QoS) is common among essential tremor (ET) patients and may have adverse effects on their quality of life, but the etiology driving the poor QoS in these individuals remains inadequately understood. The authors perform graph theory and network-based statistical analyses on ET patients with various degrees of QoS. They show that the SleET and NorET groups exhibited changes in certain graph metrics. The SleET group presented reduced nodal degrees and nodal efficiency in the right SFGmed relative to the NorET and HC groups. The observed impaired topographical organizations of functional brain networks within the central executive network (CEN), default mode network, and visual network serve to further knowledge of the complex interactions between tremor and sleep, adding to the understanding of the underlying neural mechanisms of ET with poor QoS.

*A Riemannian revisiting of structure–function mapping based on eigenmodes by* *Deslauriers-Gauthier et al*.

Understanding the link between brain structure and function may lead to better quantification of pathology. Functional connectivity matrices live in the Riemannian manifold and a specific attention must be paid to operate on this appropriate space. The authors investigate the implications of using a distance based on an affine invariant Riemannian metric. They revisit previously proposed structure–function mappings based on eigen decomposition using this adapted notion of distance. They show that using Riemannian distance alters the notion of similarity between subjects from functional perspective. They show that using this distance improves the correlation between the structural and functional similarity of subjects. Lastly, they demonstrate the importance of mapping function from structure under the Riemannian manifold and show that it is possible to outperform the group average on the performance of mappings based on eigenmodes.

## Author contributions

All authors listed have made a substantial, direct, and intellectual contribution to the work and approved it for publication.

## Conflict of interest

The authors declare that the research was conducted in the absence of any commercial or financial relationships that could be construed as a potential conflict of interest.

## Publisher's note

All claims expressed in this article are solely those of the authors and do not necessarily represent those of their affiliated organizations, or those of the publisher, the editors and the reviewers. Any product that may be evaluated in this article, or claim that may be made by its manufacturer, is not guaranteed or endorsed by the publisher.

